# Evaluation and Optimization of Prolonged Release Mucoadhesive Tablets of Dexamethasone for Wound Healing: In Vitro–In Vivo Profiling in Healthy Volunteers

**DOI:** 10.3390/pharmaceutics14040807

**Published:** 2022-04-07

**Authors:** Qurrat ul Ain Javed, Muhammad Ali Syed, Rabia Arshad, Abbas Rahdar, Muhammad Irfan, Syed Atif Raza, Gul Shahnaz, Sana Hanif, Ana M. Díez-Pascual

**Affiliations:** 1 Department of Pharmaceutics, Faculty of Pharmacy, The University of Lahore, Lahore 54770, Pakistan; quratul.ain@osa.uol.edu.pk (Q.u.A.J.); ma.pharmacist@hotmail.com (M.A.S.); rabia.arshad@bs.qau.edu.pk (R.A.); 2 Department of Physics, University of Zabol, Zabol P.O. Box 98613-35856, Iran; a.rahdarnanophysics@gmail.com; 3 Department of Pharmaceutics, Faculty of Pharmaceutical Sciences, Government College University, Faisalabad 38000, Pakistan; manipharma@yahoo.co.uk; 4 Punjab University College of Pharmacy, University of The Punjab, Lahore 54590, Pakistan; raza.pharmacy@pu.edu.pk; 5 Department of Pharmacy, Quaid i Azam University, Islamabad 45320, Pakistan; gshahnaz@qau.edu.pk; 6 Faculty of Pharmacy, The University of Sargodha, Sargodha 40162, Pakistan; 7 Universidad de Alcalá, Facultad de Ciencias, Departamento de Química Analítica, Química Física e Ingeniería Química, Ctra. Madrid-Barcelona, Km. 33.6, 28805 Alcalá de Henares, Madrid, Spain

**Keywords:** dexamethasone, hemostatic, wound healing, salivary pharmacokinetic, mucoadhesive buccal tablet, in vitro–in vivo, volunteer study

## Abstract

The aim of the projected study was to design and develop a novel strategy for evaluating the mucoadhesive potential of polymeric tablets of dexamethasone (DXM) for local delivery against wounds. Therefore, formulations (Q1–Q7) were synthesized via direct compression method by varying the concentrations of polymers, i.e., ethyl cellulose (EC) and agar extract (AG). Moreover, the mucoadhesive polymeric tablets were characterized via physicochemical, in vitro, ex vivo and in vivo experiments. However, physicochemical characteristics such as FTIR showed no interaction with different polymeric combination. Surface pH of all formulations was normal to slightly alkaline. Highest hydration of up to 6.22% and swelling index was comprehended with maximum concentration of AG (50% of total tablet weight). Whereas, ex vivo and in vivo residence time and mucoadhesion were attributed to the increased concentrations of polymers. Moreover, Q7, (optimized formulation), containing 10% of EC and 40% of AG, exhibited maximum release of DXM (100%) over 8 h, along with sufficient mucoadhesive strength up to 11.73 g, following first-order kinetics having r^2^ value of 0.9778. Hemostatic effects and epithelialization for triggering and promoting wound healing were highly pronounced in cases of Q7. Furthermore, in vivo residence time was 7.84 h followed by salivary drug concentration (4.2 µg/mL). However, mucoadhesive buccal tablets showed stability for 6 months, thus following the standardization (ICH-Iva) stability zone. In summary, DXM mucoadhesive tablets seem to be an ideal candidate for eradication of wound infections via local targeted delivery.

## 1. Introduction

Wound healing is a series of intricate biological processes that is triggered via vascular constriction originated through wounded and traumatized vessels followed by myogenic spasmodic contractions, localized autacoids factors and nerve reflexes [1]. Wounds are associated with infections resulting in increased wound exudates comprehending nutritional proteins for bacteria which further helps in their proliferation, thus delaying healing, and results in improper collagen deposition [2]. Infectious wounds impose serious complications leading to high morbidity and mortality. There are also some risk factors which obstruct and complicate the healing process [3]. Wound infection is detrimental to wound healing, but the diagnosis and management are controversial and vary among clinicians. Consequently, the prime objective of wound management is to restore the host–bacteria balance by ensuring that the wound is cleared from cell debris and microbes [4]. First-line therapy for wound infections includes antibiotics, among which cephalosporin is the first drug of choice. However, due to cephalosporin’s allergic reaction issues, the drug line switches to vancomycin, metronidazole, clindamycin and aminoglycosides. However, the main disadvantage of oral antibiotics is non-compliance, economic burden, and lack of solubility and mucoadhesion. [5] Chronic wounds are difficult to heal because of heavy bleeding and increased microbial load [6,7], and this alarming situation highly evoke the need for ideal topical targeted local delivery [8]. Therefore, a novel strategy for evaluating the mucoadhesive potential of polymeric tablets of dexamethasone (DXM) for local delivery against wounds was developed and evaluated [9]. Glucocorticoid drugs such as DXM show potent anti-inflammatory effects [10], thus being helpful in triggering the healing of wounds and improved contractile function of the muscle [11,12]. Therefore, formulations (Q1–Q7) were synthesized via a direct compression method by varying the concentrations of polymers, i.e., ethyl cellulose (EC), a sustained releasing agent [13] and agar extract (AG). Moreover, the mucoadhesive polymeric tablets were characterized via physicochemical, in vitro, ex vivo and in vivo experiments [14]. This study also aimed at improving the physiochemical and therapeutic features of DXM tablets within the polymeric matrix, providing better strength and swelling behavior [15]. The impact of polymeric blend on the mucoadhesion, strength, coagulation and release profile was investigated in vitro with great superiority compared to other groups. Moreover, the wound healing potential of the DXM mucoadhesive tablets was evaluated in wound model induced in rabbit following the standard guidelines to clearly establish the superiority of the newly developed formulation.

## 2. Materials and Methods

### 2.1. Materials

Dexamethasone phosphate (DXM), agar extract (AG), magnesium stearate and polysucralose were acquired from Hoover Pharmaceuticals^®^ (Burlington, MA, USA) on kind basis. Ethyl cellulose (EC), polyvinyl pyrollidone K30 (PVP) and lactose were obtained from Sigma Aldrich^®^ (St. Louis, MO, USA). All other reagents/solvents used in the study were used as received. Similarly, double-distilled water was used throughout the study unless specified.

### 2.2. Methodology

#### 2.2.1. Formulation Design

Formulations were prepared with the concentration of both polymers ranged between 10 to 50%, *w*/*w* as composite mucoadhesive tablets (Table 1). Currently, seven different sustained release formulations were directly compressed with a bulk of 200 mg per tablet and considering the dose of DXM as 8 mg to release up to 12 h [15]. Polyvinyl pyrollidone was selected as directly compressible binder; whereas, magnesium stearate and polysucralose were included for lubrication and sweetener role, respectively. All the ingredients were added according to the quantities listed in Table 1.

#### 2.2.2. Compression Technique

Briefly, fixed additives as well as variable amounts of the ingredients were weighed accordingly (Table 1). Thereafter, for approximately 5 min, all the ingredients were geometrically blended with the help of a small pestle and mortar. The diluent was introduced at the end of mixing. This polymeric blend was transferred to a manually driven ZP-35 rotating tablet machine with a previously lubricated die cavity. By direct compression, it was then compacted into tablets by applying a force of 2.8 tons for 3 s using an 8 mm flat-faced punch [16].

### 2.3. Solid-State Characterization

The solid-state study was conducted on the physical mixture of the optimized formulation and in the same concentration as present in the master formulae.

#### 2.3.1. Fourier Transform Infrared Analysis (FTIR)

Briefly, the drug, the polymers as well as the physical mixture of the optimized formulation were collected for infrared spectral analysis. Then, about 10 mg of the sample was taken for spectral analysis using Bruker^®^ Alpha Platinum-ATR in transmission mode and scanned in the range of 4000–600 cm^−1^ (Bruker, Billerica, MA, USA). The samples were evaluated for characteristic and identifiable peaks as any unusual peak was observed in the physical mixture [17].

#### 2.3.2. Differential Scanning Calorimetry (DSC)

The DSC analysis was carried out on the drug samples, the polymers and their mixture. Approximately, 10 mg of the sample was placed in the aluminum cup that was finally fixed with the lid plate and positioned in the analytical chamber of DSC TL Q2000TM machine (TA Instruments, New Castle, DE, USA). Temperature conditions were increased at a rate of 20 °C/min and the temperature was scanned in the range of 40 to 250 °C. The nitrogen gas was purged in the system at a rate of 50 mL/min [18].

### 2.4. Physicochemical Characterization

#### 2.4.1. Weight Variation

The individual weights of 20 tablets were precisely calculated in order to determine the average weight of the sample using a class A sensitive digital balance, and presented in terms of deviation using Equation (1) as follows:

(1) $$\mathrm{Deviation}\;\left(\%\right)=\frac{\mathrm{Individual}\;\mathrm{weight}-\mathrm{Average}\;\mathrm{weight}}{\mathrm{Average}\;\mathrm{weight}}\times 100$$

The USP standards were used for the evaluation of allowed limits [19].

#### 2.4.2. Thickness and Diameter

Both thickness and diameter of the formulations were calculated with the aid of digital Vernier caliper. Ten tablets from each batch were evaluated [20].

#### 2.4.3. Hardness

The force required to crush or crack the tablet was evaluated using automated digital hardness tester Curio HT-901. To execute, ten tablets from each formulation were selected and the crushing force was calculated for each batch [21].

#### 2.4.4. Friability

The Roche friabilator was assessed for the estimation of friability test of each formulation separately. Briefly, a sample with weight equivalent to 6.6 g was taken and placed in the friabilator chamber at 25 rpm for 4 min. After falling from the chamber height, tablets were dedusted and reweighed for estimated loss. The friability was calculated according to the formula [22], as provided by United States Pharmacopeia (USP).

#### 2.4.5. Surface pH

Surface pH of the prepared tablets was checked to evaluate the acidic or basic surface of the formulation exposed to the buccal mucosa. Concisely, tablets were allowed to swell in 10 mL of phosphate buffer solution (BPS) adjusted to pH 6.8 for 2 h at room temperature, as reported [19]. Surface pH was calculated by touching the electrode of pH meter to the surface of the tablet and allowing it to equilibrate [20].

#### 2.4.6. Swelling Index (SI)

To execute, tablets were weighed initially (W1) and kept in a petri dish over a glass slide containing 20 mL of distilled water adjusted to pH 6.8 in a way that half of the tablet remained dipped in the BPS [23]. At defined intervals, the weight gained (W2) by the tablets due to swelling was assessed using Equation (2).

(2) $$\mathrm{Swelling}\;\mathrm{Index}\;\left(\%\right)=\frac{W2-W1}{W1}\times 100$$

#### 2.4.7. Matrix Erosion (ME)

The tablet that was swelled previously during the SI was exposed to the drastic conditions of 60 °C for 24 h in an oven in order to lose ample moisture. This was followed by placement in a desiccator for 48 h prior to reweighed (W3). Then, ME was calculated using Equation (3).

(3) $$\mathrm{Matrix}\;\mathrm{Erosion}\;\left(\%\right)=\frac{W1-W3}{W1}\times 100$$

#### 2.4.8. Ex Vivo Mucoadhesive Time (ET)

The ET was measured using constructed conditions, as reported by Hanif et al. 2021. For execution, freshly excised rabbit’s buccal mucosa was removed and attached on a glass slide. For this procedure, ethical approval was taken from the Institutional Review Board of the University of Lahore under application number IREC-2019-125C. It was then immediately placed in a beaker containing 900 mL of the PBS, pH 6.8 media at an angle of 45°. Before immersing in the medium, the tablet to be tested was placed on to the surface of the mucosa by applying a force of 2.5 tons for 20 s and fixed. The whole assembly was kept and maintained at 37 °C with a stirring speed of 150 rpm. The time at which the tablet was separated or eroded from the mucosal surface was as ET [21].

#### 2.4.9. Ex Vivo Mucoadhesion Strength (MS)

##### Development of MS Apparatus

The simple physical balance was slightly modified with the intention of measuring the MS was developed using rabbit buccal mucosa was used as a model membrane with the same ethical approval [20]. Briefly, one arm was modified to measure the detachment force of the tablet from mucosa. A fixed glass slide on base with a moveable slide that was tied to the moveable balance arm in such a way that when weight was added on the other arm of the balance, the tablet was detached. The weight required to separate the glass slide from either surface of the tablet was considered as the MS value [21].

##### Evaluation of MS

When the whole set was stable and static, water drops were added on left side of the pan as weight. The weight of water (grams) required to detach the tablet from the mucosal layer was recorded as respective MS.

#### 2.4.10. Mucoadhesive Study in Volunteers (MT)

Optionally, the MT was performed on drug-free tablets by applying the tablets on the inner cheeks of healthy volunteer (gums) in order to estimate the residence time. Before the start of the experiment, favorable opinion under approval number REC/DPP/FOP/6F from the Institutional Research Ethics Committee (IREC) of The University of Lahore. Progressively, each tablet was placed on internal upper gum facing front was placed by applying very gentle force with sterile fingertip for 20 s. Then, the time was noted at which the tablet disappeared from the application point due to erosion. The MT was performed on five healthy volunteers who consented to participating in the study. Volunteers (m/f, 20–25 y) were not allowed to eat during the experiment, but they were allowed to drink. The volunteers were also inquired to report any issue pertaining to the presence of dosage form in the buccal cavity. They were allowed to report any symptoms of redness, swelling, irritation or pain associated with the application of dosage form.

### 2.5. In Vitro Drug Release Study

The in vitro dissolution study of DXM was conducted using USP paddle apparatus type II. Three tablets from each formulation code were placed in dissolution beaker separately containing 900 mL of BPS solution pH 6.8. The apparatus was maintained at 37 ± 0.5 °C with a rotation speed of paddles at 50 rpm. Samples of 5 mL were withdrawn at predetermined time intervals (0.5–12 h) with the equal volume replaced with fresh medium. Aliquot was filtered and directly run on ultraviolet Shimadzou^®^ spectrophotometer 1800 (Kyoto, Japan) for quantitative estimation at a wavelength of 241 nm for percent DXM release [24].

### 2.6. Salivary DXM Release in Volunteers

Determination of salivary drug concentration was performed only on the optimized formulation. For sampling, five human volunteers within the group of 20–25-year-olds were including for the salivary estimation of DXM. Sampling was performed on optimized formulation by applying tablets to healthy volunteers’ gums. All subjects were instructed not to take water and food starting from half an hour prior to the study. During the time interval, any possible irritation, bad taste, dry mouth or excessive salivation was also evaluated. However, it is generally reported here and will be presented as a separate study. Approximately fifteen minutes before the time interval, the volunteers were forbidden to drink anything and sample of saliva (500 μL) was collected with the help of micropipette at 0.5–12 h. Salivary sample was then diluted with 4.5 mL of acetonitrile and shaken gently for a minute. If needed, the mixture was further centrifuged for 8000 rpm for 10 min under ambient conditions. The supernatant liquid was carefully removed and the remaining portion was syringe-filtered (Parenteral grade). The filtered solution was then analyzed for drug concentration at a wavelength of 241 nm using Shimadzou^®^ spectrophotometer against the blank of salivary sample with no drug.

### 2.7. Salivary Pharmacokinetic Estimation

Model independent approach was used to estimate different pharmacokinetic parameters such as the area under the curve (AUC), maximum drug concentration, time for such concentration, extrapolated AUC, the significance of AUC and elimination rate. Microsoft Excel version 19 and GraphPad Prism^®^ version 8.0.3 (GraphPad Software, Inc., San Diego, CA, USA) was used for the calculation and construction of graphs.

### 2.8. In Vitro Salivary Release Kinetics

DD solver^®^ was applied on the in vitro release results in order to determine the mode of release of drug in optimized formulation. Kinetic modeling was applied on in vitro drug release results of optimized formulation. These models were Zero-order, First-order, Hixson–Crowell, Higuchi and Korsmeyer–Peppas model.

### 2.9. Coagulation Analysis via Optical Density

Wound bleeding coagulation capability can be determined via coagulation analysis. Therefore, for this purpose, fresh human blood was collected with the consent of volunteers in the anti-coagulant vials. Furthermore, DXM tablets were then immersed in the blood followed by incubation at 37 °C for 30 min. However, optical density was determined to analyze the release of free hemoglobin from red blood cell (RBC) membrane lyses at 540 nm [25].

### 2.10. In Vivo Histopathological Analysis

Animal experimentation for determining histopathological evaluation was performed after the favorable approval of the Institutional Review Board of The University of Lahore under application number IREC-2019-125C. Therefore, 6 groups of rabbits (*n* = 3) were kept with free access of food and water at suitable environment. Therefore, left maxillary cleft of the rabbit lip was fixed, followed by minor wound creation, and the rabbit mucosa was exposed to treatment and healing was observed for 5 days. Moreover, rabbit mucosa was observed on daily basis. Afterwards, controlled, wounded and treated mucosal tissues were removed, followed by fixation in the buffered formalin solution at ambient temperature for 4 h. Furthermore, all the extracted tissues were embedded in paraffin for sectioning of tissues in various directions. The extracted sections were then stained with the hematoxylin and eosin (H&E) for further microscopic evaluation via imaging [26].

### 2.11. Stability Study

The optimized formulation was subjected to stability study conditions up to the time of 6 months where intermittent sampling was performed to evaluate the performance of the formulation. The compressed formulation was sealed inside aluminum foil and placed in the stability chamber according to International Conference for Harmonization (ICH) guidelines—zone Iva, i.e., 40 °C with relative humidity (RH) of 75% [27]. During each testing interval, MS and ET were evaluated according to the methodology reported earlier. For content uniformity, briefly, ten tablets were crushed finely in a pestle and mortar. Then the weight equivalent to 200 mg was taken and added in a beaker containing 900 mL of PBS pH 6.8. It was stirred magnetically at 800 rpm for 45 min. Then, 5 mL of the aliquot was removed and filtered using syringe filter and evaluated for quantitative estimation of DXM.

### 2.12. Statistical Analysis

The in vitro release profile of the optimized dosage form was additionally performed after the end of stability and was compared with the release data of the optimized formulation (before stability) for the determination similarity (*f*_2_) and dissimilarity factors (*f*_1_). Moreover, Student’s *t*-test was performed on the release data of the optimized formulation before and after stability to find whether the difference between the means of release profile values really exists or not [28].

## 3. Results and Discussion

With the aim of producing a sustained release dosage form of DXM to reduce inflammation and healing of the ulcerative conditions, different physicochemical tests were employed on the prepared mucoadhesive formulations. The physicochemical testing was optimized in order to select a formulation which can be used to produce a sustained action in the buccal cavity of volunteers. As the buccal mucoadhesive route was selected, it was intended to maintain a prolonged therapeutic concentration of DXM in the buccal cavity. Different batches of buccal mucoadhesive formulations of master study (Q1–Q7), were prepared with variable concentration of the polymers. Evaluation of the prepared formulations was performed via different testing parameters and results were recorded and are described in the following section. Formulations were evaluated for physicochemical parameters and mucoadhesion properties, as well. As far as the master study is concerned, formulation Q2, Q3, Q4, Q5 and Q6 contained a polymeric blend of EC and AG in increasing and decreasing trends, whereas Q1 and Q7 contained single polymer only, so that properties of single polymers could also be revealed in terms of their in vitro release and mucoadhesive properties.

### 3.1. Solid-State Characterization

#### 3.1.1. FTIR

**Figure 1:**
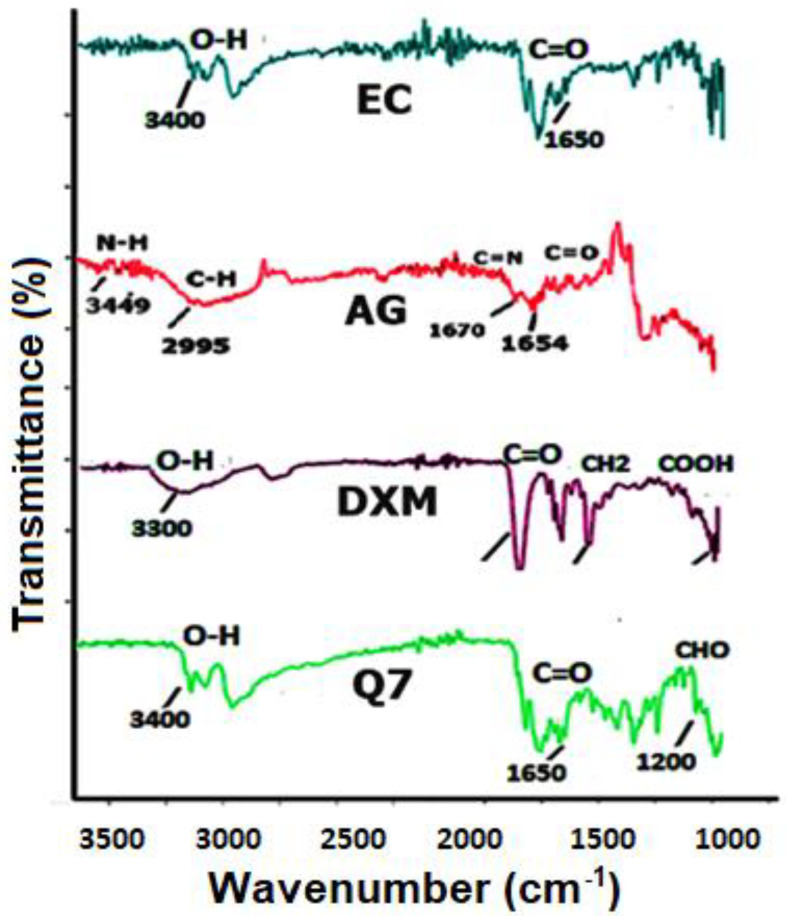
FTIR spectra of DXM, the polymer and the physical mixtures.

FTIR peaks of pure DXM, the polymers (EC and AG) and the physical mixture (Q2) were analyzed, and no abnormal peaks were observed in any of the spectrum (Figure 1). EC showed a strong absorption band at 1650–1550 cm^−1^ ascribed to the C=O stretching vibration [29], while the O-H stretching of primary amine group occurred at 3400–3100 cm^−1^. On the other hand, the FTIR spectrum of pure DXM showed significant bands at 3500–3300 cm^−1^, 3000–2700 cm^−1^ and 1600 cm^−1^, which corresponded to O–H stretching, aromatic C–H stretching and C=O stretching, respectively [30]. The main absorption peaks of AG were observed at 3300–2500 cm^−1^, depicting –OH stretching, 1780–1650 cm^−1^ (C=O of carbonyl group), 3300–2700 cm^−1^ (C–H bond), 1450–1375 cm^−1^ (C–H bending of CH_2_) and 1300–1100 cm^−1^ due to the asymmetric stretching of C–O–H [31,32]. Thus, the characteristic peaks of the ingredients were present in the Q7 of the optimized formulation, and the absence of unusual peaks is confirmed.

#### 3.1.2. DSC

**Figure 2:**
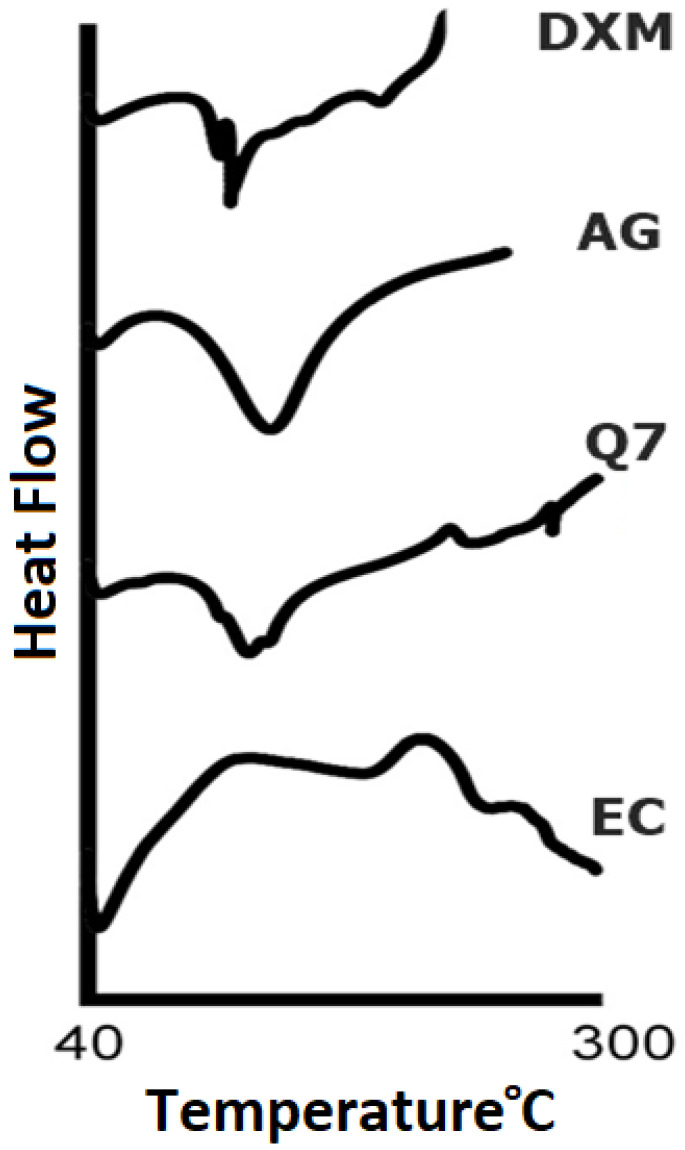
DSC thermograms of DXM and polymer and physical mixtures.

The endothermic curve of the physical mixture according to optimized formulation revealed sharp peaks at the point of drug, indicating that the crystalline structure of DXM and the polymers were preserved in the compressed form. Second endothermic peaks were observed at 300 °C, as shown in Figure 2. First endothermic peaks represented the dehydration process, and second endothermic peak was attributed to degradation under atmospheric nitrogen.

### 3.2. Physical Characterization

**Figure 3:**
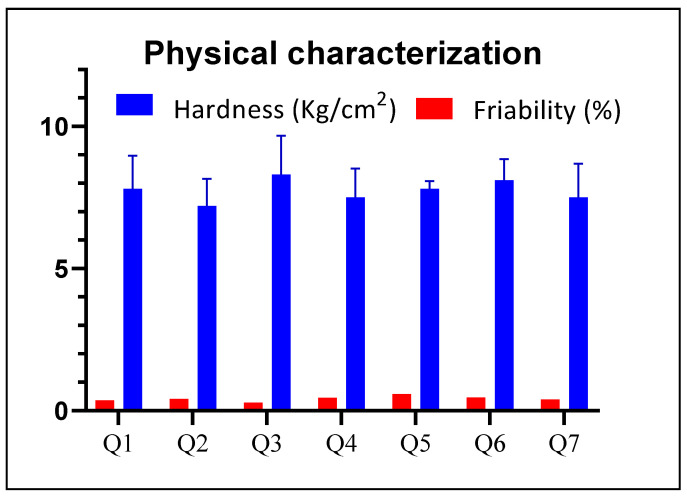
Comparison of the physical parameters of buccal formulations.

All the prepared mucoadhesive formulations were evaluated for physical characterization. Physical parameters included weight variation, thickness, diameter, hardness and friability. Results of all physical tests were found to be within the limits. Weight of the tablet was found in the range of 198 mg to 201 mg with an acceptable deviation. According to USP, for the tablet weight of 200 mg, 7.5% weight variation is allowed. Minimum weight was found with Q7 that was 198.2 mg, while maximum weight of 203.5 mg was found with Q5. Hardness was set in the range of 7–9 kg/cm^2^ (Figure 3), whereas, the least hardness was found with Q2, i.e., 7.2 and maximum hardness was given by Q3, i.e., 8.3. Thickness of mucoadhesive tablets was within the range of 3.75 mm to 3.77 mm with minimum standard deviation of 0.01, whereas the diameter of tablets was found in the range of 8.03 mm to 8.05 mm with the standard deviation of 0.01. Friability was in range according to standards of USP which was less than 1% *w/w* [33]. It is noticed that variable concentration of polymers did not cause the physical testing to fall behind the limits and all test results complied with the standard limits, as shown in the Table 2.

### 3.3. Surface pH

The pH of all formulations (Q1–Q7) was found in the range of 7.31 to 7.60. Although there has been very slight variation in the findings reported by a previous researcher, it is in the physiological range according to that study [34]. Maximum pH was found with the formulation Q5, i.e., 7.6, while minimum value was observed with the formulation Q1, i.e., 7.31. Therefore, the pH of formulations was found to be in a slightly basic range. Pathology is supposed to occur when the pH differences are significant or larger as compared with the physiological pH of the buccal cavity.

### 3.4. Swelling Index

**Figure 4:**
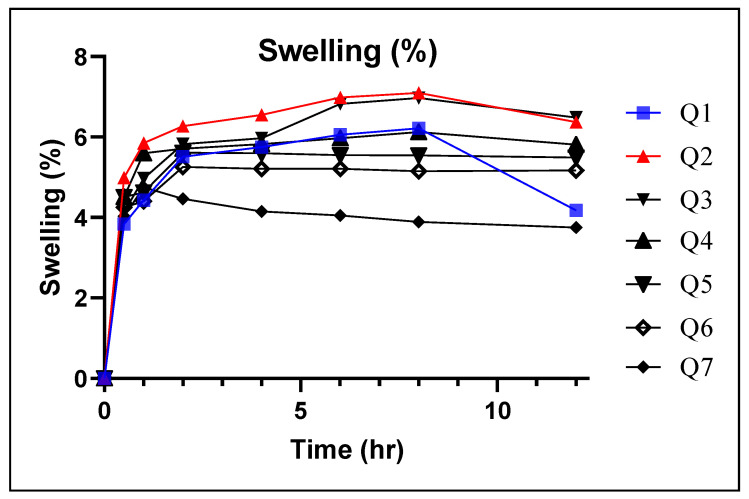
Swelling index of compressed tablet composites.

Swelling index depicts the extent of water absorption by the tablets from the media which in turn will swell to form a three-dimensional matrix network. Ultimately, the release of the active medicament will then be retarded from this polymeric network. Results revealed an increase in the tablet weight and a swelling of the diameter as a function of time. Among all formulations, Q2 exhibited the maximum swelling as it contained 40% AG and 10% EC. Regarding the rest of the formulations, a slight but notable increase was observed with each formulation, and it was shown that high swelling was associated with a high concentration of AG, while less swelling was observed for the formulations containing even high amounts of EC (Figure 4). Swelling is more related to the presence of AG, and this might be due to the hydration and gelling ability of this polymer. The results also depicted that the tablets swelled initially to a maximum inherent capability and then either slowly decreased as in the case of Q7 or slightly increased further, as was observed, for instance, with Q2.

**Figure 5:**
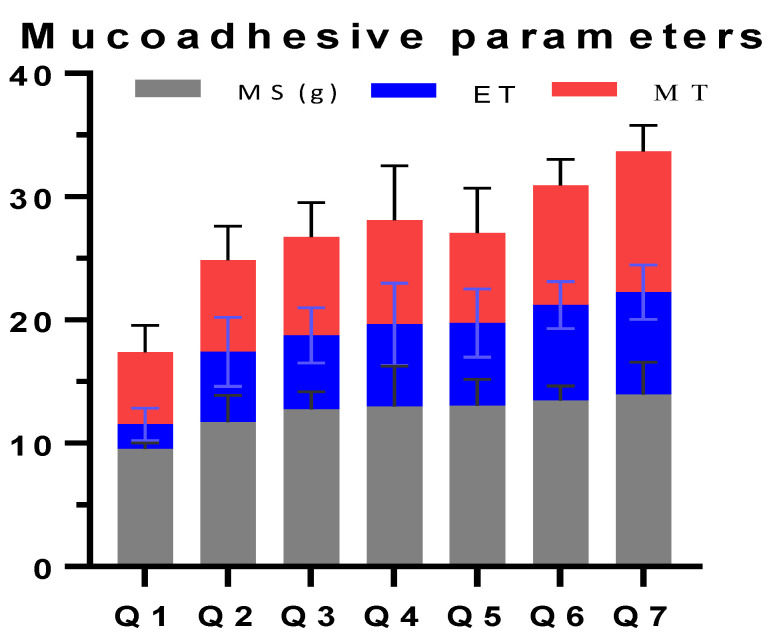
Mucoadhesive characterization of formulations prepared in the study.

Thus, the swelling index in the formulations Q1–Q7 was directly related with the concentrations of polymers: as the concentration of EC increased, the extent of swelling was reduced [22]. However, Q7 comprised a single polymer that was EC (50%) and AG (5%) showed least swelling of 3.75% at the end of 12 h. Whereas, Q1 containing AG (50%) and EC (5%) expressed swelling of tablets of up to 4.18% until 12 h. Since EC is a hydrophobic polymer due to the presence of the ethyl group, it is assumed that the poor hydration is directly linked to the hydrophobicity of the dosage form [35]. Similarly, there was no clear gel appearance around the surface of the formulations when it was in contact with the media. This parameter can be linked with the poor MS values of the formulations (Figure 5).

### 3.5. Matrix Erosion (ME)

Matrix erosion depicts the trend of dosage form degradation when it is in contact with the buccal media. It is an in vitro estimation of the fate of the dosage form after releasing the medication. It was found that the higher the concentration of EC, the lower the ME of the formulations. The largest ME value was found when EC was present in least concentration, i.e., Q1 and the ME decreased as the concentration of EC was reduced, i.e., Q7 (63.46%), as presented in Table 2.

### 3.6. Ex Vivo Mucoadhesive Time (ET)

The observed ET was found in the range of 1.98 to 8.29 h. The shortest mucoadhesive time was found for Q1 containing a single polymer that was AG (50%) and EC (5%), while maximum mucoadhesion time was observed for Q7 with AG (5% *w/w*) and EC (50% *w/w*). An increasing trend in the mucoadhesion time was observed on increasing AG concentration in Q1–Q7 (Figure 5).

### 3.7. Ex Vivo Mucoadhesion Strength

The force required to separate the tablet from mucosal surface is measured as mucoadhesive strength. The results showed a growing trend for increasing EC concentration. However, the maximum strength value was found for Q7 (13.96 g, Figure 5). As the concentration of EC increased, the MS also increased. However, it is important to note that the increase in the MS of EC from 10 to 50% *w/w* concentration did not produce a significant increase compared with hydrophilic polymers like hydroxyl propyl methyl cellulose. It, however, contributes to the lower adhesive strength of the EC which is consistent with former literature [22]. It can be explained from the hydrophilic behavior of the polymer.

### 3.8. Mucoadhesive Time in Volunteers (MT)

The MT was estimated in volunteers who were willing to participate the study and not suffering any acute form of any oral infection or pathology. Results calculated on MT for drug-free tablets were found in the range of 5.83–11.43 h (Figure 5). The least MT (5.83 h) was observed with Q1 containing single-polymer AG (50% *w/w*) and EC (5% *w/w*), while the greatest residence time of 11.43 h was reported with Q7 containing EC in maximum concentration used in the study, along with 5% *w/w* of AG. It was also noted that no tablet caused any sign of swelling, irritation, pain or discomfort to the application site. It was also observed in the cavity that the formulations did not depict any significant swelling, which is correlated with the findings of SI.

### 3.9. In Vitro Drug Release

**Figure 6:**
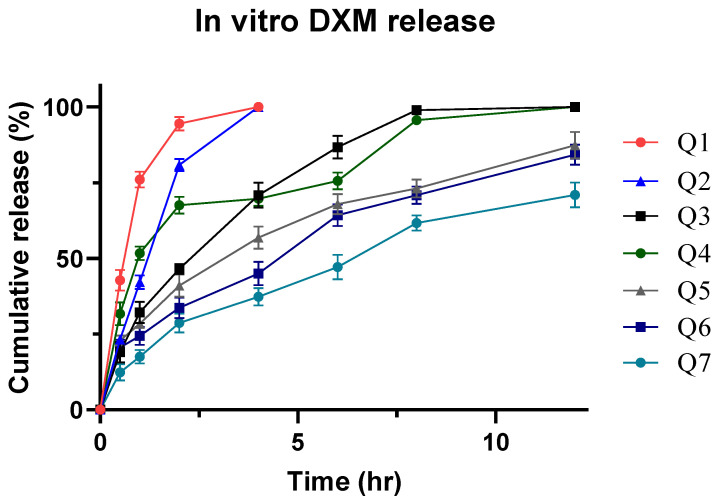
In vitro DXM release using PBS 6.8 media.

Since the quantification was performed on a UV spectrophotometer, a calibration curve was constructed for DXM to estimate the linearity range using PBS (pH 6.8). The dilutions were prepared from the stock solution of DXM in the range of 0.5–12 µg/mL which showed a linear expression of y = 14.041x − 13.041, with a regression coefficient value of 0.9990. From the dissolution study, it was clearly observed that the nature and concentration of the polymer had an influence over drug release, as it is conditioned by the capability of the polymer to contain and release the drug as a function of time, as shown in Figure 6. As the concentration of EC was increased, a shift was observed in the sustained releasing trend from formulations Q1 to Q7. The slowest release was observed with Q7 that had a value of approximately 70.91% till 12 h. Following Q7 was Q6 that released 84.16% of DXM at the end of 12 h. Regarding the other formulations, Q1 and Q2 released DXM completely within 4 h and formulations Q3 and Q4 released >99% DXM till 8 h. Initial burst release was observed with formulation Q1 that released almost 42.80% of the drug until 0.5 h. The mechanism behind sustained release of DXM is the hydrophobic behavior of EC [36] and as the concentration of the polymer is increased, the dissolution of DXM is reduced. AG seemed to play a minor role in the release of DXM as it was released in a burst manner when the concentration of AG was maximum in Q1. Based on these results, it can be inferred that Q7 is optimal in sustaining the DXM release in the mucoadhesive buccal tablets.

### 3.10. Optimization of Formulation

The optimization of the results was primarily based on sustained drug release. When in vitro release data were analyzed, it was found that formulation Q7 depicted the slowest form of drug release and displayed almost 70.91% ± 4.16 DXM release. Moreover, it exhibited better MS (13.96 g) and MT (11.43) values compared with the rest of the formulations. However, it was unable to swell as much as Q1. It was supposed to deliver sustained release of DXM during in vivo sampling in healthy volunteers. In the Q7 formulation, solid-state characterization, salivary drug concentration, stability testing, pharmacokinetic estimation and statistical analysis were applied.

### 3.11. Salivary DXM Release in Volunteers

**Figure 7:**
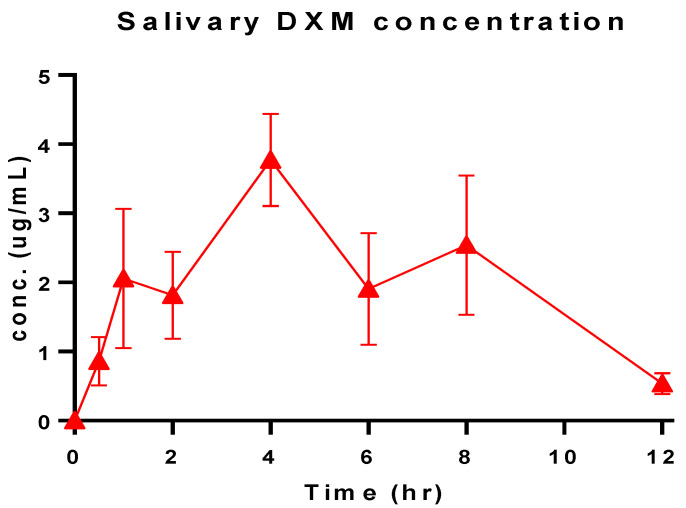
Salivary DXM concentration of the optimized formulation (Q7) over time.

**Figure 8:**
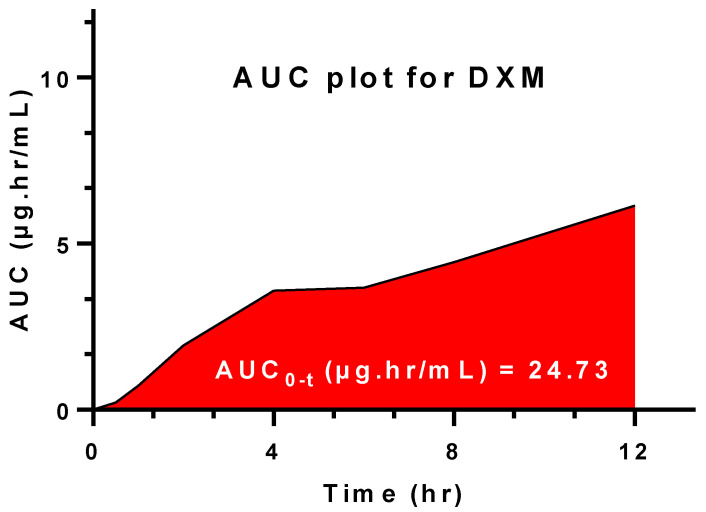
Area under the curve (AUC) from t_0–12 h_ for Q7.

To maintain effective drug concentration in saliva, it was important for the dosage form to be applied locally, otherwise the oral delivery of the DXM would have systemic adverse effects [37]. Moreover, a greater dose of the drug would in turn be required for the therapeutic effects. Mucoadhesion through buccal delivery for localized healing action was the ultimate task for this problem. The salivary drug concentration ranged between 0.536–3.771 µg/mL (Figure 7) during the time period between 0.5 and 12 h. While maximum drug concentration was found near 4 h (t_max_), at the beginning and the end of the interval, values lower than 1 µg/mL were observed. This may be due to the starting-phase DXM releasing and the exhaustive time when all the drug was released. A steady increasing AUC plot was observed until 12 h (Figure 8). The total AUC according to the model independent approach was 26.44 µg.hr/Ml, where the contribution of extrapolated AUC was insignificant. The rate of elimination of DXM form the salivary fluid was 0.31 hr^−1^ against a topical dose of 8 mg for 12 h (Table 3). [38]. A similar study compared the topical DXM concentration for local action and were less than 1 ug.hr/mL in sustained release form [39]. It suggests the effectiveness of the drug in dosage form for healing action.

### 3.12. In Vitro-Salivary Release Kinetic

It was found that the optimized formulation (Q7) followed Korsmeyer–Peppas mode of drug release since the value of r^2^ for this model was maximum compared with others. The value of *n* for the Korsmeyer–Peppas model was 0.554 with 0.45 ≤ x ≤ 0.89 [40]. This value demonstrates that the release of DXM from the dosage form was based on a diffusion plus erosion mechanism [41]. It reflects that for a longer period of time, it was difficult for the mucoadhesive tablet to withstand stressful environment due to which part of the dosage form also eroded somehow to release the drug in the buccal region [42]. The behavior of salivary DXM also followed the Korsmeyer–Peppas model (Table 4) and as the dosage form is squeezed between the gum and the inner check mucosa, it is assumed that the dosage form might have eroded slightly to release drug both through diffusion and erosion mechanism. However, the value of the regression coefficient was quite low. This was attributed to the downward salivary action which rinsed out the drug from the buccal cavity [16]. Ultimately, the cumulative drug release did not consistently increase over time, rather, the concentration of DXM ranged at a constant level with slight variation.

### 3.13. Coagulation Analysis via Optical Density

**Figure 9:**
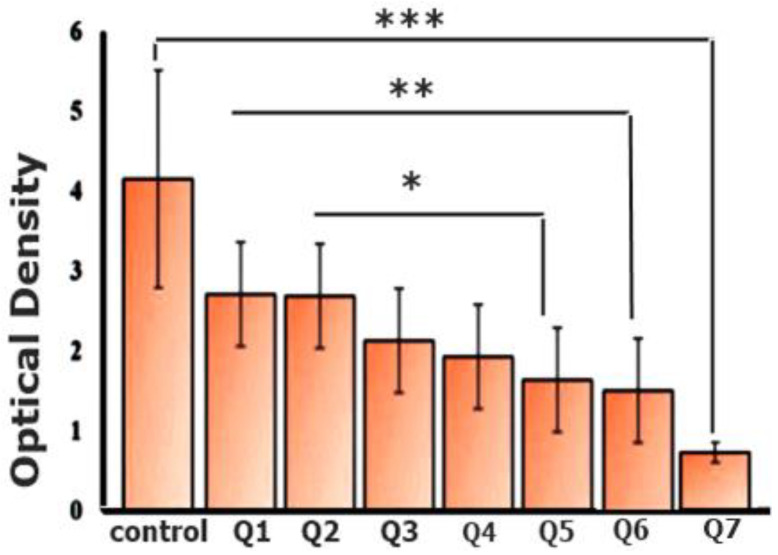
Coagulation analysis of mucoadhesive DXM tablets via optical density determination (Note that * symbol in figure represents *p* value of 0.0001 to 0.001, while ** means 0.001 to 0.01 and *** corresponds to 0.01 to 0.05).

The coagulation determination test resulted in low optical density values of Q7, i.e., 0.5, as compared to other formulations and control, as shown in Figure 9. Low optical density values are often associated with the decreased flow of free hemoglobin after the degradation of red blood cells [25]. Therefore, the results demonstrate that the final formulation Q7 can strongly act as an anticoagulant formulation, thus promoting healing [25].

### 3.14. In Vivo Histopathological Evaluation

**Figure 10:**
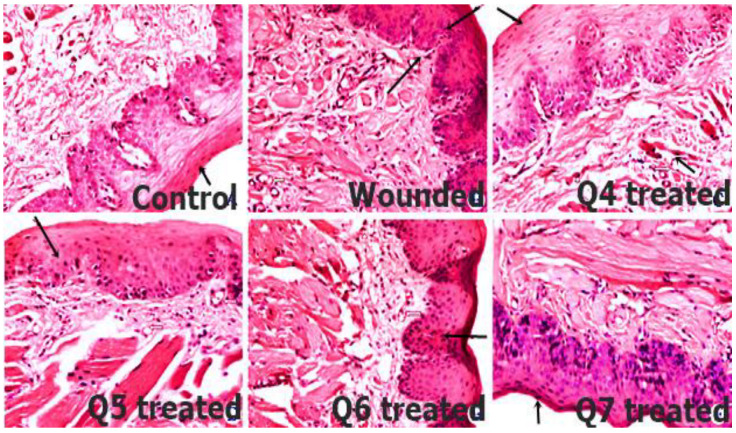
Histopathological evaluation of wounded buccal mucosal tissues by comparing control, wounded and treatment groups (Q4–Q7). Newly generated mucosal tissues with complete epithelialization were evident with Q7 treatment group at day 5.

Histopathological evaluation of wounded buccal mucosa was performed to examine the histological changes, as shown in Figure 10. Newly generated mucosal tissues with complete epithelialization were visible with Q7 after day 5. Therefore, it was evident that Q7 formulations possess strong wound healing features by targeting macrophages. Macrophages are inflammation inducers owing to the excessive release of cytokines and neutrophils. However, in the histological imaging, healing could be confirmed by mature epithelialization of mucosal tissues (Q7 treated), compared to the other formulations and control group.

### 3.15. Stability Study

**Figure 11:**
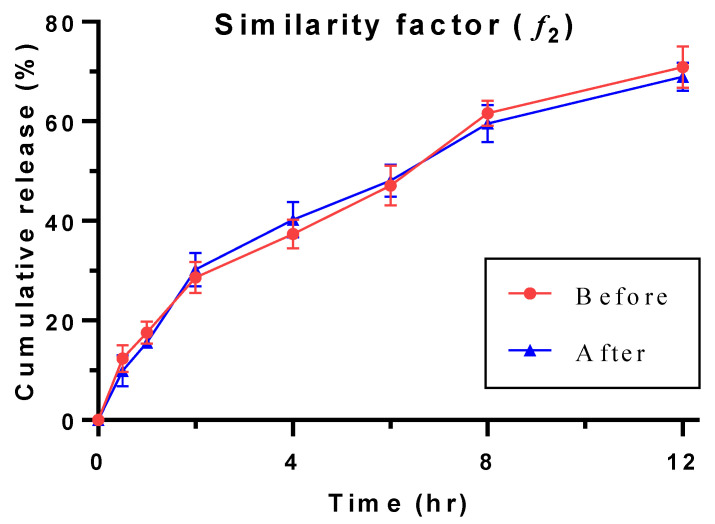
Comparison of release profiles of Q7 exposed before and after stability condition.

With the intermittent sampling analysis, it was found that the content uniformity of DXM was safely retained in the drug delivery and the least change in the drug contents and mucoadhesive parameters since no significant change in the mucoadhesive properties was found and the amount of the drug present in the tablet did not differ significantly (Figure 11). This was further confirmed with the specification values of similarity and dissimilarity factors [43]. The respective values of each test on the release of the DXM from the optimized formulation before and after stability (Table 5) were found to be within the specification [44]. Moreover, the statistical analysis on the mean values of the release profile of DXM before and after stability revealed a value of 0.608, which is considered as insignificant. It can be concluded that the difference before and after the release profile of DXM was unreal (Table 6).

## 4. Conclusions

The application of mucoadhesive drug delivery carriers has opened new avenues of advancement in wound healing, with great adaptability and targeting capability for overcoming various limitations of conventional delivery systems. Bioinspired polymers have shown promising results in treating wounds. Therefore, it is obvious that buccal mucoadhesive delivery of DXM-loaded polymeric tablets for local healing action can be an alternative option amongst conventional and technological based deliveries. Hence, the buccal mucoadhesive delivery can be an attractive alternative to produce consistent salivary drug concentration up to 12 h with a dose of 8 mg of dexamethasone. Moreover, hemostatic effects and epithelialization of tissue mucosa for triggering and promoting wound healing was highly pronounced. Furthermore, in vivo residence time was 7.84 h followed by salivary drug concentration (4.2 µg/mL), in turn, followed by stability for 6 months. The results of this research indicate the application of novel DXM polymeric mucoadhesive tablets for improving wound healing and bioavailability. Further pharmacodynamics studies on animal or human are warranted to achieve the therapeutic potential of synthesized DXM tablets for their commercialization.

## Figures and Tables

**Table 1:** Composition (%, *w*/*w*) of compressed mucoadhesive formulations.

Ingredients	Q1	Q2	Q3	Q4	Q5	Q6	Q7
DXM	4	4	4	4	4	4	4
Agar	50	40	30	25	20	10	5
EC	5	10	20	25	30	40	50
PVP	5	5	5	5	5	5	5
Poly. sucralose	2	2	2	2	2	2	2
Mg. stearate	3	3	3	3	3	3	3
Lactose	36	36	36	36	36	36	36

**Table 2:** Physical characterization of mucoadhesive tablets.

Code	Weight Variation (mg ± SD)	Diameter (mm ± SD)	Thickness (mm ± SD)	ME (%)	Surface pH
Q1	200.4 ± 1.91	8.04 ± 0.07	3.77 ± 2.08	83.74	7.31
Q2	198.9 ± 2.18	8.03 ± 0.04	3.76 ± 1.97	79.68	7.38
Q3	201.6 ± 1.52	8.05 ± 0.09	3.77 ± 0.62	77.12	7.42
Q4	200.4 ± 1.83	8.04 ± 0.16	3.76 ± 0.89	71.37	7.32
Q5	203.5 ± 1.87	8.05 ± 0.08	3.77 ± 0.21	72.36	7.60
Q6	200.1 ± 2.05	8.05 ± 0.11	3.76 ± 1.31	67.28	7.37
Q7	198.2 ± 1.99	8.03 ± 0.09	3.75 ± 0.46	63.46	7.41

**Table 3:** Salivary pharmacokinetic estimation of the optimized formulation for local release.

Parameters	Findings
Dose (mg)	8
C_max_ (µg/mL)	3.77
t_max_ (h)	4
k_el_ (h^−1^)	0.31
AUC_0–t_ (µg·hr/mL)	24.73
AUC_t–∞_ (µg·hr/mL)	1.70
AUC_0–∞_ (µg·hr/mL)	26.44
AUC_t–∞_ (%)	6.43
Contribution AUC_t–∞_	insignificant

**Table 4:** In vitro and salivary drug release kinetics of Q7 formulation.

Model	Zero Order		1st Order		Higuchi		Korsmeyer-Peppas			Hixson-Crowell	
	r^2^	k_0_	r^2^	k_1_	r^2^	k_H_	r^2^	k_KP_	*n*	r^2^	k_HC_
In vitro	0.970	7.001	0.9507	0.117	0.989	20.204	0.9931	18.206	0.554	0.9333	0.033
Salivary	0.082	2.518	0.127	0.032	0.318	8.891	0.3301	20.484	0.05	0.112	0.01

**Table 5:** Stability data of the optimized formulation under IVa regional guidelines.

Interval (Months)	Contents (% ±SD)	ET	MS
0	98.17 ± 1.44	8.29 ± 2.21	13.96 ± 2.63
0.5	99.42 ± 0.58	8.11 ± 1.42	13.73 ± 2.44
1	98.96 ± 1.36	8.19 ± 2.83	13.9 ± 1.09
3	98.01 ± 0.10	8.21 ± 2.65	13.95 ± 2.51
6	99.32 ± 0.25	8.1 ± 1.19	13.91 ± 2.18
Release profile comparison after stability conditions			
Dissimilarity factor (*f*_1_) [Specification 0–15]		5.07	
Similarity factor (*f*_2_) [Specification 50–100]		83.07	

**Table 6:** Outcome of the Student’s t-test for Q7 formulation exposed before and after stability conditions.

Before-After Stability	Mean	Standard Deviation	Standard Error Mean	95% Confidence Interval of the Difference		*t* Value	df	Sig. (2-Tailed)
				Lower	Upper			
**DXM**	0.385	2.02	0.71	−1.31	208	0.536	7	0.608

## Data Availability

The data presented in this study are available on request from the corresponding author.
